# Impact of COVID-19 on Acute Stroke Presentation at a Comprehensive Stroke Center

**DOI:** 10.3389/fneur.2020.00850

**Published:** 2020-08-14

**Authors:** Masaki Nagamine, Daniel S. Chow, Peter D. Chang, Bernadette Boden-Albala, Wengui Yu, Jennifer E. Soun

**Affiliations:** ^1^Department of Neurology, University of California, Irvine Medical Center, Orange, CA, United States; ^2^Department of Radiological Sciences, University of California, Irvine Medical Center, Orange, CA, United States; ^3^Center for Artificial Intelligence in Diagnostic Medicine, University of California, Irvine, Irvine, CA, United States; ^4^Department of Population Health and Disease Prevention, Department of Epidemiology, University of California, Irvine, Irvine, CA, United States

**Keywords:** stroke, COVID-19, public health, thrombolytics, stroke triage

## Abstract

**Background:** COVID-19 has impacted healthcare in many ways, including presentation of acute stroke. Since time-sensitive thrombolysis is essential for reducing morbidity and mortality in acute stroke, any delays due to the pandemic can have serious consequences.

**Methods:** We retrospectively reviewed the electronic medical records for patients presenting with acute ischemic stroke at a comprehensive stroke center in March–April 2020 (the early months of COVID-19) and compared to the same time period in 2019. Stroke metrics such as incidence, time to arrival, and immediate outcomes were assessed.

**Results:** There were 48 acute ischemic strokes (of which 7 were transfers) in March–April 2020 compared to 64 (of which 12 were transfers) in 2019. The average last known well to arrival time (±SD) for stroke codes was 1,041 (±1682.1) min in 2020 and 554 (±604.9) min in 2019. Of the patients presenting directly to the ED with a known last known well time, 27.8% (10/36) presented in the first 4.5 h in 2020, in contrast to 40.5% (15/37) in 2019. Patients who died comprised 10.4% of the stroke cohort in 2020 (5/48) compared to 6.3% in 2019 (4/64).

**Conclusions:** During the first 2 months of COVID-19, there were fewer overall stroke cases who presented to our hospital, and of these cases, there was delayed presentation in comparison to the same time period in 2019. Recognizing how stroke presentation may be affected by COVID-19 would allow for optimization of established stroke triage algorithms in order to ensure safe and timely delivery of stroke care during a pandemic.

## Introduction

The overwhelming burden of the current COVID-19 pandemic on the healthcare system has produced unintended consequences on acute stroke care. COVID-19 which was first diagnosed in Wuhan, China, and spread rapidly worldwide, has affected over 4.5 million individuals worldwide and over 1.4 million in the U.S. as of May 15, 2020 (1). While the number of COVID-19 cases presenting to hospitals has risen exponentially, there are reports of precipitous declines in the number of patients presenting with acute stroke in many countries, including China, Italy and the U.S. (2–5). Reports suggest a global increase in treatment delays for life threatening and disabling conditions and at worst, an increase in the number of patients presenting outside of time sensitive treatment windows (6–9). The impact of COVID-19 on outcomes of stroke patients who may be delaying or foregoing presentation to hospitals is consequential since early interventions are potentially life-saving. Time-sensitive thrombolysis for qualifying ischemic stroke patients is a critical component of the stroke treatment paradigm. Tissue plasminogen activator (tPA) up to 3 to 4.5 h and thrombectomy up to 24 h have been established as the standard of care and have shown therapeutic benefit in large randomized clinical trials (10–12). The purpose of this study is to evaluate our institution's experience with patients presenting with acute stroke symptoms during the early months of the COVID-19 pandemic.

## Methods

We retrospectively reviewed patients who presented with acute stroke symptoms to University of California Irvine Medical Center (UCI) during the time period of March 1–April 30, 2020. This period was chosen to coincide with our hospital's initial experience with COVID-19. UCI is a 411-bed comprehensive stroke center serving Orange County, CA, with 317 acute ischemic stroke cases who presented in 2019. We analyzed the incidence, time to arrival, severity, administered therapies, and immediate outcomes of stroke cases during the early months of COVID-19 and compared to the same time period (March 1–April 30, 2019) 1 year prior. All data was obtained through UCI's stroke database and electronic medical record, and the study was performed in accordance with IRB guidelines.

## Results

A total of 48 patients presented with acute stroke symptoms during March 1-April 30, 2020, in comparison to 64 patients from March 1–April 30, 2019 (Table 1). This included 1 patient with COVID-19 diagnosed by nucleic acid detection test. Mean age was 65.25 ± 16.1 years in 2020 compared to 69 ± 14.9 years in 2019. There were 7 interfacility transfers (IFT) and 3 inpatient strokes in 2020 and 12 IFTs and 3 inpatient strokes in 2019. The mean time from last known well (LKW) to arrival for stroke code patients was 1,041 ± 1682.1 min in 2020 and 554 ± 604.9 min in 2019. Of the patients presenting directly to the ED with a known last known well time, 27.8% (10/36) presented in the first 4.5 h in 2020, in contrast to 40.5% (15/37) in 2019. The mean presenting NIH Stroke Scale (NIHSS) score was 10.29 ± 8.5 and discharge NIHSS if the patient survived was 5.86 ± 5.2 in 2020 in comparison to 9.52 ± 9.8 and 7.05 ± 8.4, respectively, in 2019. Five patients each received tPA in 2020 and 2019, and 9 patients underwent thrombectomy in 2020 in contrast to 4 patients in 2019. Patients who died comprised 10.4% of the stroke cohort in 2020 (5/48) compared to 6.3% in 2019 (4/64). Mean time from patient arrival to administration of tPA (door to needle) in minutes was 49.6 (±37.2) in 2020 compared to 50 (±20.1) in 2019. Mean time from patient arrival to vessel puncture for endovascular therapy (door to puncture) in minutes was 109 (±32.4) in 2020 compared to 132 (±22.6) in 2019.

**Table 1:** Demographics, time metrics, and early clinical outcomes of acute ischemic stroke patients in March–April 2020 (COVID-19 pandemic) in comparison to March–April 2019.

	**March–April 2020 (*n* = 48)**	**March–April 2019 (*n* = 64)**
Age, y, mean ± SD	65.25 ± 16.1	69 ± 14.9
Female, *n* (%)	12/48 (25%)	25/64 (39%)
**Race**		
Hispanic	20	11
Black/African-American	0	3
Non-Hispanic White	12	42
Asian	14	8
**Stroke presentation**, ***n***		
Stroke code or consult	36	37
Inpatient stroke	3	3
Interfacility Transfer	7	12
Unknown LKW	2	12
LKW to Arrival in minutes, mean ± SD^*^	1,041 ± 1682.1	554 ± 604.9
Presentation <4.5Hr in minutes, *n* (%)	10/36 (27.8%)	15/37 (40.5%)
Door to imaging in minutes, mean ± SD	25.35 ± 32.8	18 ± 11.6
Door to needle in minutes, mean ± SD	49.6 ± 37.2	50 ± 20.1
Door to puncture in minutes, mean ± SD	109 ± 32.4	132 ± 22.6
NIHSS at presentation, mean ± SD	10.29 ± 8.5	9.52 ± 9.8
NIHSS at discharge if patient survived, mean ± SD	5.86 ± 5.2	7.05 ± 8.4
**Thrombolysis**, ***n***		
tPA	5	5
Thrombectomy	9	4
**TICI**, ***n***		
0, 1, or 2a	1	1
2b, 2c, or 3	8	3
Deaths, *n* (%)	5 (10%)	4 (6%)

* *Excludes inpatient strokes, IFT's, and unknown LKW*.

*LKW, last known well; IFT, interfacility transfer; NIHSS, NIH Stroke Scale; tPA, tissue plasminogen activator; TICI, thrombolysis in cerebral infarction*.

## Discussion

The COVID-19 pandemic has affected the presentation of acute stroke cases in our single-center experience during the early months of the pandemic. We found a decreased number of stroke cases and a delayed presentation to the hospital in 2020 in comparison to 2019. In addition, fewer cases were presenting in the acute time window when tPA could be administered for qualifying patients, although more patients underwent thrombectomy which suggests increased numbers of stroke cases from large vessel occlusion. Despite additional regulations instated for donning personal protective equipment during stroke code presentations in 2020, the door-to-needle and door-to-puncture times were similar between the 2 years. Interestingly, the initial NIHSS was similar between the two time periods, and the discharge NIHSS was actually better in 2020. These findings warrant further investigation, but improved discharge NIHSS may be related to higher rate of thrombectomy. However, a greater percentage of patients died from stroke in 2020 in comparison to 2019.

Changes in stroke presentation due to COVID-19 may be a result of several factors. Patients may be reluctant to seek healthcare for fear of being exposed to the virus and presenting only when strokes are severe. In addition, due to restrictions in public gatherings and social distancing measures, patients who have incapacitating strokes tend to be found by others in a delayed fashion prior to presentation. Delays in presentation prevent patients from meeting criteria for life-saving therapies and may cause increased morbidity and mortality given the time-sensitivity of treatments. Patients with milder strokes or transient ischemic attacks could be avoiding the healthcare system altogether, which could be dangerous since these patients are at increased risk of a recurrent ischemic stroke and warrant secondary prevention (13).

In order to ensure continued safe and timely acute stroke management during COVID-19, updated stroke algorithms have been proposed that address all steps of the stroke management pathway, including pre-hospitalization and interfacility transfer, hospitalization and treatment, and discharge and rehabilitation (14–16). These guidelines have introduced measures such as COVID-19 screening and personal protective equipment into stroke triage to ensure the safety of patients and the stroke team while emphasizing the importance of preventing delays in care. The optimization of telemedicine and other virtual clinician guidance tools has become essential to triage patients appropriately and provide education and prevention (17).

Limitations of this study include a retrospective single-center experience, which may not be generalizable to other stroke centers. Patient presentation to surrounding stroke centers are not accounted for in this study. Additionally, the patient number is low, allowing for only descriptive analysis of the statistics. Only immediate clinical status has been reported, and long-term clinical outcomes remain to be seen. The effect of COVID-19 on delays once the patient reaches the hospital also needs to be assessed to see if current protocols need further optimization.

In conclusion, this study highlights how stroke presentation has been impacted by COVID-19, with fewer overall cases, delays in presentation, and increased mortality. To address these potential issues, updated stroke guidelines which incorporate adherence to COVID-19 precautions into triage and treatment algorithms will allow patients to continue to receive optimal stroke care. Furthermore, more public outreach focusing on awareness of time-sensitive treatment windows for cerebral reperfusion may help prevent delays in presentation and irreversible brain injury during COVID-19 and future pandemics.

## Data Availability Statement

The raw data supporting the conclusions of this article will be made available by the authors, without undue reservation.

## Ethics Statement

The studies involving human participants were reviewed and approved by the Institutional Review Board, Human Research Protections at the Office of Research, University of California, Irvine. Written informed consent for participation was not required for this study in accordance with the national legislation and the institutional requirements.

## Author Contributions

MN collected, analyzed, interpreted the data, and helped to write the manuscript. JS wrote the manuscript. DC, PC, WY, BB-A, and MN were major contributors in the study design and editing of the manuscript. All authors read and approved the final manuscript.

## Conflict of Interest

The authors declare that the research was conducted in the absence of any commercial or financial relationships that could be construed as a potential conflict of interest.

## References

[1] Dong E Du H Gardner L. An interactive web-based dashboard to track covid-19 in real time. Lancet Infect Dis. (2020) 20:533–4. 10.1016/S1473-3099(20)30120-1 32087114 PMC7159018

[2] Zhao J Rudd A Liu R. Challenges and potential solutions of stroke care during the coronavirus disease 2019 (covid-19) outbreak. Stroke. (2020). 51:1356–7. 10.1161/STROKEAHA.120.029701 32228369 PMC7219852

[3] Morelli N Rota E Terracciano C Immovilli P Spallazzi M Colombi D. The baffling case of ischemic stroke disappearance from the casualty department in the covid-19 era. Eur Neurol. (2020) 83:213–15. 10.1159/000507666 32289789 PMC7179532

[4] Kansagra AP Goyal MS Hamilton S Albers GW. Collateral effect of covid-19 on stroke evaluation in the united states. N Engl J Med. (2020) 383:400–1. 10.1056/NEJMc2014816 32383831 PMC7233187

[5] Baracchini C Pieroni A Viaro F Cianci V Cattelan AM Tiberio I. Acute stroke management pathway during coronavirus-19 pandemic. Neurol Sci. (2020) 41:1003–5. 10.1007/s10072-020-04375-9 32270359 PMC7141930

[6] Markus HS Brainin M. Covid-19 and stroke—a global world stroke organization perspective. Int J Stroke. (2020) 2020:1747493020923472. 10.1177/1747493020923472 32310017 PMC11927026

[7] Feral-Pierssens AL Claret PG Chouihed T. Collateral damage of the covid-19 outbreak: expression of concern. Eur J Emerg Med. (2020) 27:233–4. 10.1097/MEJ.0000000000000717 32345850 PMC7202126

[8] Hecht N Wessels L Werft F-O Schneider UC Czabanka M Vajkoczy P. Need for ensuring care for neuro-emergencies—lessons learned from the covid-19 pandemic. Acta Neurochir. (2020) 162:1795–801. 10.2139/ssrn.3605279 32514620 PMC7276655

[9] Montaner J Barragán-Prieto A Pérez-Sánchez S Escudero-Martínez I Moniche F Sánchez-Miura JA. Break in the stroke chain of survival due to covid-19. Stroke. (2020) 51:2307–14. 10.1161/STROKEAHA.120.030106 32466738 PMC7282408

[10] Hacke W Kaste M Bluhmki E Brozman M Davalos A Guidetti D. Thrombolysis with alteplase 3 to 4.5 hours after acute ischemic stroke. N Engl J Med. (2008) 359:1317–29. 10.1056/NEJMoa0804656 18815396

[11] Albers GW Marks MP Kemp S Christensen S Tsai JP Ortega-Gutierrez S. Thrombectomy for stroke at 6 to 16 hours with selection by perfusion imaging. N Engl J Med. (2018) 378:708–18. 10.1056/NEJMoa1713973 29364767 PMC6590673

[12] Nogueira RG Jadhav AP Haussen DC Bonafe A Budzik RF Bhuva P. Thrombectomy 6 to 24 hours after stroke with a mismatch between deficit and infarct. N Engl J Med. (2017) 378:11–21. 10.1056/NEJMoa1706442 29129157

[13] Amarenco P Five-year risk of stroke after tia or minor ischemic stroke. N Engl J Med. (2018) 379:1580–81. 10.1056/NEJMc1808913 30332572

[14] Dafer RM Osteraas ND Biller J. Acute stroke care in the coronavirus disease 2019 pandemic. J Stroke Cerebrovasc Dis. (2020) 29:104881. 10.1016/j.jstrokecerebrovasdis.2020.104881 32334918 PMC7164903

[15] Fraser JF Arthur AS Chen M Levitt M Mocco J Albuquerque FC. Society of neurointerventional surgery recommendations for the care of emergent neurointerventional patients in the setting of covid-19. J NeuroInterv Surg. (2020) 12:539–41. 10.1136/neurintsurg-2020-016098 32295835

[16] Khosravani H Rajendram P Notario L Chapman Martin G Menon Bijoy K Protected code stroke. Stroke. (2020) 51:1891–5. 10.1161/STROKEAHA.120.029838 32233980 PMC7258750

[17] Sheth Sunil A Wu T-C Sharrief A Ankrom C Grotta James C Fisher M. Early lessons from world war covid. Stroke. (2020) 51:2268–72. 10.1161/STROKEAHA.120.030154 32421392 PMC7258749

